# Utility of ER, p53, CEA and Napsin A in Histological Subtyping of Endometrial Carcinoma and Their Correlation with Clinicopathological Prognostic Parameters: Experience from a Referral Institute

**DOI:** 10.30699/IJP.2024.2008693.3154

**Published:** 2024-01-29

**Authors:** Saumya Shivakumar, Kausalya K Sahu, Ranjitha Rao, Chaithra GV, Cheryl Sarah Philipose, Sharada Rai

**Affiliations:** 1 *All India Institute of Medical Sciences, Bhopal, India*; 2 *Department of Pathology, Kasturba Medical College, Mangalore, Manipal Academy of Higher Education, Manipal, India*

**Keywords:** Cancer, Carcinoma, CEA, Endometrial, ER, Napsin A, p53

## Abstract

**Background & Objective:**

Endometrial Carcinoma (EC) is the most common gynecological cancer with a global incidence of 23.2 per 1 lakh population. Histological subclassification of EC is extremely crucial for the diagnosis, proper management strategies, and prognosis. This study was conducted in a tertiary care institute to analyze the expression pattern of a minimum panel of 4 markers (ER, p53, CEA, Napsin A) with emphasis on their utility in the routine histological subtyping, aberrant expression, and correlation with various clinicopathological parameters.

**Methods:**

A time-bound cross-sectional observational and analytical study was conducted, which includes cases diagnosed in our laboratory from January 2016 to April 2021.

**Results:**

Sixty cases diagnosed as EC during the study period formed the sample cases. The ER was expressed in 85% (53/60) of cases in the current study. Among them, 94% (50/53) were endometrioid endometrial carcinomas (EECs). A negative correlation was found between ER intensity and age (r= -1.48). Of 60 EC cases, 10 (16%) cases expressed p53. The tumors positive for p53 with higher intensity were negative for ER and vice versa. The expression pattern of ER and p53 was statistically significant (*P*=-0.021). On IHC, 84.6% (11/13) of CEA-positive cases expressed both ER and CEA, suggesting mucinous differentiation. Napsin A was expressed in two cases of EEC, FIGO grade I, and one case of serous carcinoma.

**Conclusion:**

An inverse association was found between ER and p53 expression. The CEA is valuable in identifying EEC with mucinous differentiation.

## Introduction

Endometrial carcinoma (EC) is the most common gynecological cancer with a global incidence of 23.2 per 1 lakh population (1-3). In India, the EC accounts for 11.50% of female genital tract cancers, which is next to cervical and ovarian cancer (4). Bokhman identified two distinct forms (types I and II) of EC. In premenopausal and postmenopausal women, type I tumors are the most common and are linked to unopposed estrogenic stimulation and endometrial hyperplasia. Type II carcinomas are hormone-independent and evolve primarily in postmenopausal women's atrophic endometrium (5). This categorization suggests distinct etiopathogenesis and biological behaviors of these tumors (3). Type I tumors include endometrioid endometrial carcinoma (EEC) and mucinous carcinomas. These are classified as grade 1, 2, or 3 by the International Federation of Gynecology and Obstetrics (FIGO) based on glandular differentiation. Type II cancers include serous Carcinoma (SC), mixed mullerian tumor (MMT), and clear cell carcinoma (CCC). These carry a bad prognosis as they often present at the advanced stage and are considered as higher grades irrespective of histology (6).

Thus, histological subclassification of endometrial carcinoma is extremely crucial for the diagnosis, proper management strategies, and prognosis. It is difficult to exactly subtype the EC on morphology alone as features overlap especially in high-grade tumors. Though immunohistochemistry (IHC) is a useful, adjunctive tool in this regard, it often requires a panel of markers. They also showed ambiguity in a few cases with nonspecific and aberrant expression of markers (3).

This study was conducted in a tertiary care referral institute to analyze the expression patterns of a minimum panel of 4 markers in EC i.e. ER, p53, CEA, and Napsin A with emphasis on their utility in the routine histological subtyping, aberrant expression, and correlation with various clinicopathological parameters including grading and staging.

## Material and Methods

This time-bound cross-sectional observational and analytical study includes cases diagnosed in our laboratory from January 2016 to April 2021. Institutional ethical clearance was obtained for the study. All histologically confirmed cases of EC (hysterectomy only) were included. The slides and paraffin blocks of the cases were retrieved. For the prospective cases, hysterectomy specimens diagnosed preoperatively as EC were collected. Gross features noted. Tumor sections were embedded in paraffin blocks. Tissue sections were stained with Hematoxylin and Eosin (H&E) as per the standard operating procedure. 

The slides were reviewed by two independent pathologists. Clinicopathological parameters were noted. The relevant clinical data was collected from the registers in the computer data systems.

**IHC Staining and Analysis**

Monoclonal mouse anti-human ER antibody clone 1D5 and ER-2-123 (Ig G) in tissue culture supernatant, Tris HCL buffer, stabilizing protein, and 0.015 mol/L of sodium azide was used for the IHC of ER. For the p53 detection, rabbit anti-human p53 monoclonal antibody in 10 mm PBS, at pH 7.4, 0.2% BSA, and 0.09 % sodium azide was used. Clone SP5 Rabbit IgG monoclonal mouse antibody in 0.05 mol/L Tris-HCl buffer, pH 7.2, with 15 mmol/L NaN_3 _was used for the CEA detection. For the Napsin A detection monoclonal mouse antibody provided in Tris Buffer at pH 7.3-7.7, along with 1% BSA and Sodium Azide <0.1% was used. The staining protocol was performed according to the manufacturer’s instructions.

For the ER marker analysis, tumor cells with only distinct nuclear staining were considered as positive. Interpretation of the ER expression was done using the formula: PS (percentage of stained cells) + IS (intensity of stain) = TS (total score) (7). The percentage of tumor cells stained positive were graded as 0, 1, 2, 3, 4, and 5 for the absent staining, ≤1% staining, 2–10% staining, 11–33% staining, 34–66% staining, and 67–100% staining, respectively. The staining intensity of tumor cells was graded 0, 1, 2, and 3 for no staining, weak, intermediate, and strong staining, respectively. Out of a TS of 8, a score of 2 or more was considered as positive.

Tumor cells with only distinct nuclear staining were considered p53 positive. Ten random fields were selected and percentage positivity of p53 expression and intensity of staining (with x400 objectives) were noted. The stainings were graded as follows: intensity 0: negative, 1: weakly positive, 2: moderately positive, and 3: strongly positive. The percentage scores were graded as 0, 1, 2, and 3 for <5% positive, 5-25% positive cells, 25-75% positive cells, and >75% positive cells, respectively. The total score was calculated by adding the PS and IS. It was expressed as – for score 0, + score 1-2, ++ for score 3-4, and +++ for score 5- 6 (8).

For the CEA, luminal membrane staining was considered as positive, and the staining was assessed semi-quantitatively depending on the intensity and extent of the staining (9). The intensity score (IS) is quantified as 0, 1, 2, and 3 for the negative staining, weak, moderate, and strongly positive staining, respectively. The percentage score (PS) was given as 0, 1, 2, and 3 for 0%, 1 to 10% reactivity, 11 to 50% reactivity, 51 to 80% reactivity, and 81 to 100% reactivity, respectively. The total score (TS) was calculated by multiplying IS and PS. A score of 1 or more was considered positive and a score of 0 as negative. 

For the Napsin A, granular cytoplasmic positivity in tumor cells was considered positive. For the statistical analyses, the intensity of the staining was graded as 0, 1+, 2+, and 3+ for the negative, weak, moderate, and strong staining, respectively. The results were classified into four groups based on the intensity and percentage of tumor cells showing Napsin A positivity as follows: weakly positive (≤70% of cells with 1+ intensity or ≤30% of cells with 2+ intensity), moderately positive (>70% of cells with 1+ intensity or 31–70% of cells with 2+ intensity in or ≤30 %, and cells with 3+ intensity) and strongly positive (> 70% of cells with 2+ intensity or >30% of cells with 3+ intensity) (10).

SSPS software version 22.0 (SPSS Inc., Chicago, Ill., USA) was used for statistical analysis. The correlation of IHC markers expression (ER, p53, CEA, and Napsin A) with clinicohistopathological parameters was analyzed with the Chi-square test and Fisher’s exact test. The significant level was determined based on the P-value.

## Results

Sixty cases of hysterectomy specimens, which were diagnosed as EC on histopathology during the study period formed the sample cases for the study. The mean and median ages of the patients were 56 ± 11.7 and 57 ± 10.5 years, respectively. The EEC was 90% (54/60) of total EC, followed by 5% cases (3/60) of SC and MMT each.

**IHC Analysis of EC Cases**

The ER, p53, CEA, and Napsin A expression in all 60 cases of EC were studied with the histomorphology, grading, staging, and various clinicopathological parameters as given in Table 1.

**Table 1:** Correlation between expression of ER, p53, CEA, Napsin A markers, and various clinicopathological parameters.

Clinicopathological features	ER		p53		CEA		Napsin A	
Age groups	P	N	P	N	P	N	P	N
31-40	100% (6)	0% (0)	16.6% (1)	83.3% (5)	33.33% (2)	66.6% (4)	0% (0)	100% (6)
41-50	91.6% (11)	8.33% (1)	8.33% (1)	91.6% (11)	25% (3)	75% (9)	8.33% (1)	91.6% (11)
51-60	85.7% (12)	14.2% (2)	0% (0)	100% (14)	21.4% (3)	78.57% (11)	0% (0)	100% (14)
61-70	85% (17)	15% (3)	30% (6)	70% (14)	25% (5)	70% (15)	5 % (1)	95% (19)
71-80	87.5% (7)	12.5% (1)	12.5 (2)	87.5% (6)	0% (0)	100% (8)	12.5(1)	87.5% (7)
Total	53	7	10	50	13	47	3	57
Tumor size								
<5cms	95.57% (45)	4.42% (2)	11.11% (5)	88.88% (40)	20% (10)	80% (37)	4.2% (2)	95.7% (45)
>5cms	61.53% (8)	38.46% (5)	38.5% (5)	61.5% (8)	23.1% (3)	76.9% (10)	7.69% (1)	92.3% (12)
			10	50	13	47	3	57
Histological type								
Endometrioid	92.59% (50)	7.4% (4)	9.4% (5)	90.6% (49)	22.2% (12)	77.8% (42)	3.7% (2)	96.3% (52)
Serous	66.66% (2)	33.33% (1)	66.66% (2)	33.33% (1)	33.3% (1)	66.66% (2)	33.3% (1)	66.66% (2)
MMT	33.33% (1)	66.66% (2)	100% (3)	0% (0)	0% (0)	100% (3)	0% (0)	100% (3)
			10	50	13	47	3	57
FIGO Stage								
1A	82.85% (29)	17.14% (6)	11.4% (4)	88.6% (31)	14.3% (5)	85.7% (30)	8.6% (3)	91.4% (32)
1B	91.66% (11)	8.3% (1)	16.7% (2)	83.3% (10)	25% (3)	75% (9)	0% (0)	100% (12)
2	100% (8)	0% (0)	37.5% (3)	62.5% (5)	37.5% (3)	62.5% (5)	0% (0)	100% (8)
3A	100% (4)	0% (0)	20% (1)	80% (4)	50% (2)	50% (2)	0% (0)	100% (4)
3B	100% (1)	0% (0)	0% (0)	100% (0)	0% (0)	100% (1)	0% (0)	0% (1)
			10	50	13	47	5	57
FIGO GRADING								
1	93.61% (44)	6.3% (3)	2	45	6.3% (9)	93.6% (38)	4.3% (2)	95.7% (45)
2	83.33% (5)	16.6% (1)	2	4	16.6% (2)	83.3% (4)	0% (0)	100% (6)
3(7 cases- one EEC, 3 SC and 3 MMT)	57.1% (4)	42.8% (3)	6	1	42.8% (2)	57.1% (5)	14.3% (1)	85.7% (6)
total	53	7	10	50	13	47	3	57
Myometrial invasion								
Less than 50%	100% (31)	0% (0)	6.5% (2)	93.5% (29)	19.4% (6)	80.6% (25)	6.5% (2)	93.5% (29)
More than 50%	75.86% (22)	24.13% (7)	27.6% (8)	72.4% (21)	24.13% (7)	75.86% (22)	3.4% (1)	96.6% (28)
total	53	7	10	50	13	47	3	7
LUS involvement (3 cases)	33.3% (1)	66.6% (2)	0% (0)	100% (3)	0% (0)	100% (3)	0% (0)	100% (3)
Cervical involvement (5 cases)	100% (5)	0% (0)	20% (1/5)	80% (4)	60% (3)	40% (2)	0% (0)	100% (5)
Parametrial involvement (3 cases)	100% (3)	0% (0)	0%(o)	100% (3)	66.66% (2)	33.3% (1)	0% (0)	100% (3)
Lymph node metastasis (2 cases)	100% (2)	0% (0)	50% (1)	50% (1)	50% (1)	50% (1)	0% (0)	100% (2)
Lymphovascular invasion (11 cases)	90.9% (10)	9.9% (1)	27.3% (3)	72.7% (8)	36.4%(4)	63.6% (7)	18.2% (2)	81.81% (9)

P: Positive, N: Negative

**ER Expression**

**Fig. 1:**
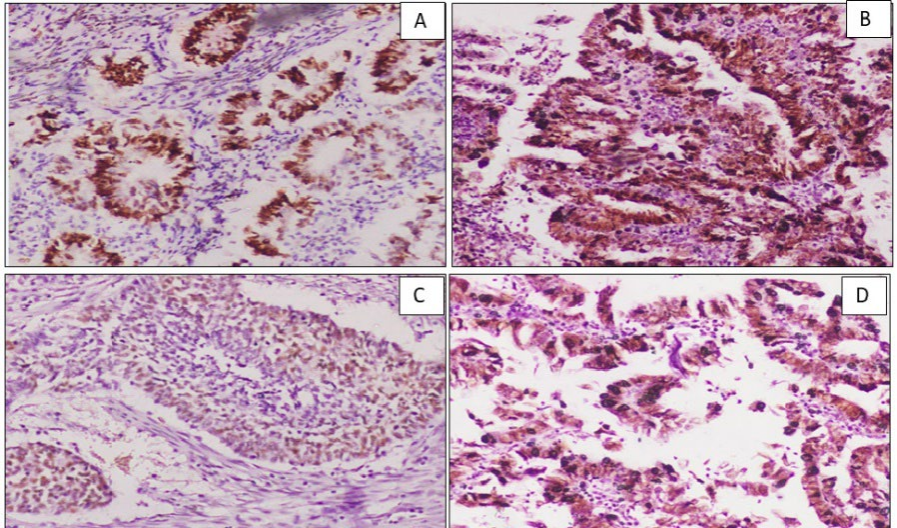
A.Photomicrograph showing strong ER positivity (3+) in the tumor cells in grade I EEC. (10X) B. Photomicrograph showing the aberrant expression pattern of p53 in the serous carcinoma. (10x). C. Photomicrograph showing wild-type expression of p53 in grade III EEC (10X). D Aberrant expression of p53 in a case of grade III EEC (10x).

The ER was expressed in 85% (53/60) of cases (Figure 1A). In that 94% (50/53) were EECs, 3.9% (3/53) were SC, and 1.9% (1/53) were MMT (*P=*0.196). Four EEC cases were ER-negative. 

 The majority of the cases positive for the ER were in the age range of 40-60 years. A negative correlation was found between ER intensity and age (r=- 1.48), which means the intensity of the ER expression was stronger in younger women. On correlating tumor size and ER expression (cut-off taken as 5 cm in the largest dimension), 95.57% (45/47 cases with size less than 5 cm) showed ER expression with a score >2. In tumors with a size more than 5 cm, 61.53% (8/13 cases) showed ER expression >2. However, these findings were not statistically significant (*P*=0.996).

Maximum numbers of cases were positive for the ER in the stages IA (82.85%, 29/35) and IB (92%, 11/12). Statistically, no significant difference was observed (*P*=0.196). Also, the ER was expressed in most of the cases of LG EECs as compared to the HG cases. However, no significant level could be obtained (*P*=0.423). 

Of four ER-negative EEC cases, two (50%) were FIGO grade I and showed negativity for p53, CEA, and Napsin A. One ER-negative case was low grade with CEA positivity and without cervical involvement. However, p16 detection was not performed in this case. The other ER-negative case was FIGO grade III with focal p53 positivity.

**P53 Expression**

Of 60 cases of EC, 10 (16%) expressed p53. The expression of p53 was seen predominantly in age group 61 to 70 years and it was statistically significant (*P*=0.038). Also, p53 showed positive correlation with size (*P*=0.009). Only 9.4% (5/53) of EEC cases showed positivity compared to 33.33% (1/3) and 100% (3/3) positivity in serous (Figure 1B) and MMTs, respectively. These findings were statistically significant (*P*=0.001) (Table 2). Further, all MMT cases showed the highest positivity score (3+), whereas EECs showed predominantly 2+ positivity (Figure 1C). In SC, one case showed 3+ positivity and the remaining two cases showed complete negativity. Interestingly, one EEC case, which was FIGO grade I but advanced stage (stage IIIA with LUS, cervix, and lymph node involvement) showed strong p53 positivity (score 3+) (Figure 1D). Also, this case showed focal ER and CEA positivity. These findings were statistically significant (*P*=0.001)

For the p53 cases, out of 10 positive cases, 90% (9/10) were in the lower stage (stage I-6/10, stage II-3/10) compared to the advanced stage (stage III-1/10, stage IV-0 cases) and no statistical significance was observed (*P*=0.123). The grade I tumors had low positivity (2/47, 4.2%) compared to the grade II (2/6, 33.3%) and the highest positivity was seen in grade III tumors (6/7, 85.7%). A statistically significant correlation was observed between p53 expression and FIGO grading (*P*<0.001).

Other clinicopathological parameters (LUS involvement, cervix involvement, lymph node metastasis, etc.) did not show any significant correlation with the expression of p53 (*P*>0.05).

**Table 2:** P53 expression scores in various histological types.

Serial number	histological type	p53 expression (PS+IS)	Total score
1	EEC	2+2=4	++
2	EEC	2+1=3	+
3	EEC	3+2=5	++
4	MMT	3+3=6	+++
5	MMT	3+3=6	+++
6	EEC	1+3=4	++
7	MMT	4+3=7	+++
8	EEC	2+3=5	+
9	SC	3+3=6	+++
10	EEC	3+3=6	+++

**ER and p53 Co-expression**

Of 60 cases, 13% (8/60) expressed both ER and p53 markers. Out of 54 EECs, five cases (9.2%) showed both ER and p53 expression. All 3 cases of SC expressed ER along with p53. The tumors that were positive for p53 with higher intensity were negative for the ER and vice versa. The expression pattern of ER and p53 was statistically significant (*P*=0.021). 

**CEA**

Of 60 EC cases, 22% (13/60) expressed CEA as luminal membranous staining. Of that 92.3% (12/13) were EEC and one case was SC histologically (Table 3). No significant correlation was observed between CEA expression and other clinicopathological parameters (*P*>0.05).

**Fig. 2:**
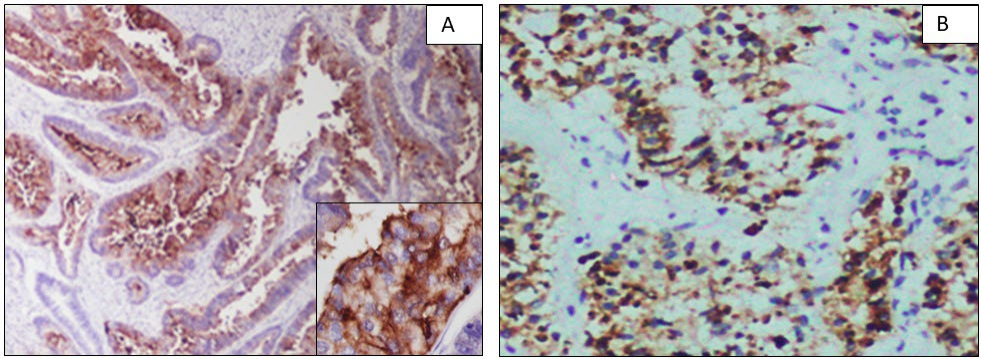
A. Luminal membrane expression of CEA suggesting mucinous differentiation in a case histopathologically diagnosed as EEC. (10x) Inset (40x). B. A strong cytoplasmic positivity of Napsin A in a case of EEC. (10x)

On IHC detection, 84.6% (11/13) of CEA-positive cases expressed both ER and CEA suggesting mucinous differentiation (Figure 2A). All of these cases on histology showed glandular differentiation and were called EEC on histology. They were the majority of grade I (72.7%, 8/11) followed by grade II (18.18%, 2/11). However, no statistical significance was observed between the ER and CEA co-expression and grade (*P*=0.201). Amongst the CEA-positive cases, the majority were of FIGO stages I and II. One case that showed CEA expression but was negative for the ER expression was in grade I and stage IA tumor with no LUS or cervical involvement. One SC that showed CEA positivity was grade 3 and stage IB with lymphovascular invasion. Interestingly. This also had focal positivity for the Napsin A and ER.

**Table 3:** Details of the cases that showed CEA expression. (PS: Percentage score, IS: intensity score)

Serial number	Histo type	FIGO grade	CEA IS X PS	ER PS+IS	P53 PS+IS
1	EEC	2	1X3=3	3+2=5	0+0=0
2	EEC	1	2X3=6	3+3=6	0+0=0
3	EEC	1	2x3=6	3+3=6	2+1=3
4	EEC	3	1X2=2	3+3=6	0+0=0
5	EEC	2	1x3=3	3+2=5	3+2=5
6	EEC	1	1X2=2	3+3=6	0+0=0
7	EEC	1	3X3=9	1+2=3	0+0=0
8	EEC	1	1X3=3	2+1=3	0+0=0
9	SC	3	2X3=6	1+1=2	3+3=6
10	EEC	1	2X3=6	3+3=6	0+0=0
11	EEC	1	3x3=9	4+2=6	0+0=0
12	EEC	1	1X3=3	2+1=3	3+3=6

**Napsin A Expression**

Napsin A was expressed in 5% of the EC cases (3/60). Of that, 66.66% (2/3) were morphologically EEC, FIGO grade I and one was serous carcinoma. All cases also showed ER positivity. One EEC that showed 3+ positivity was FIGO grade I and stage I (Figure 2B).

## Discussion

The risk stratification (low or high risk) of newly diagnosed EC cases was traditionally done based on the FIGO stage, histologic subtype, and grade. The stratification was very crucial as it would guide the management in terms of extensive versus limited surgery and the need for adjuvant therapy. Recently, molecular markers such as ER, p53, PR, and Her 2/neu status were also proposed. They not only help in identifying the histologic typing of cases but also provide the scope for the targeted therapy (11).

An inverse correlation was found in the present study between ER intensity and age (r = -1.48) that showed the intensity of ER expression was stronger in younger women. This also correlates with Bokhman’s classification of type I EC, which is estrogen-dependent and is seen in premenopausal age groups. The majority of the positive cases for the ER were in the age range of 40-60 years. Similar findings were observed in other studies (12,13). ER was expressed in most cases of LG (FIGO I &II) tumors compared to the grade III in our study similar to other studies (13-18). A significant association was documented in the previous studies between ER/PR expression and clinicopathological parameters (endometrioid histology, lower grade, or absent/minimal myometrial invasion) (19,20). Shivkumar* et al.*, and Wang* et al.* also found a significant association between the FIGO stage and ER expression (16,14). However, no significant correlation was observed between ER with grade and stage in our study (*P*=0.423).

Four cases with grade I EEC morphology were ER-negative. Guan J* et al.* (21) in their study observed that EEC cases with ER/PR loss had a greater risk of myometrial invasion, higher stage, lymph node metastasis, and higher relapse rate compared to the ER/PR positive group. The ER receptor assessment is also a pre-requisite for the patients’ selection for the treatment with tamoxifen (ER modulator) and aromatase inhibitors, which are tried in advanced and recurrent cases (22).

The p53 immunohistochemistry has been identified as a surrogate marker reflecting the underlying TP53 mutation status of a tumor. Stavropoulos* et al.* stated that p53 can be introduced as a diagnostic tool of importance in high-grade cancers, especially serous carcinomas. It can be used for differentiating serous carcinomas from endometrioid carcinomas when the histomorphological architecture is blurred (8). In the present study, 17% (10/60) of EC cases expressed p53. Amongst these 50% (5/10) were formed by grade III EEC (1/10), 30% by MMT (3/10), and 20% by SC (2/10). A very high significant level was seen in the correlation of MMTs and p53 expression since all 3 cases of MMTs showed strong positivity towards p53 in our study. This finding was concordant with the studies conducted by Schwarz* et al.* and Reid* et al.* (6, 18). 

Previous studies have observed that overexpression of p53 in EC is a poor prognostic factor and mostly it correlates with less differentiation, higher stage, myometrial invasion, lymph node, and distant metastases (23-26). In our study, p53 was significantly correlated with high-grade tumors, which is concordantly seen in the studies by Shivkumar* et al.*, (16), Stavropoulos* et al.* (8), and Salama* et al.* (1). In the present study, p53 also showed significantly high positive correlation with age, which infers that higher age group (60-80 years) are more likely to have p53 expression and higher grade. The same findings were also observed in other studies (6, 8, 16, 18, 27, 28). The p53 also showed a positive correlation with size. In our study, tumor stage had no significant correlation with p53, this was discordant with Shivkumar* et al.* (16) and Daniilidou K,* et al.* (29) studies where they found higher p53 expression in the advanced-stage tumors. Stavropoulos* et al.* (8) in their study observed a significant correlation between the p53 staining score and the histologic subtypes and fallopian tube or ovarian invasion (*P*=0.014), whereas no such correlation was seen in our study (*P*˃0.05).

Interestingly, Stavropoulos* et al.* (8) also observed that the statistical analysis showed significant results when the sum of the p53 score (including both intensity and percentage positivity) and clinicopathological features were compared and the results were insignificant when only intensity or percentage positivity was analyzed alone with the other parameters.

In SC, 75% (2/3) of cases showed complete negative p53 staining (score 0). In tubo-ovarian serous carcinomas, the 3-tier system of p53 interpretation was followed, wherein it is classified as aberrant expression and wild-type expression. Aberrant expression also known as abnormal expression (complete positive/complete negative nuclear staining in >80% tumor cells) correlates with underlying TP53 mutation whereas wild-type expression (weak staining/mosaic nuclear staining <80% tumor cells) is seen in normal tissues (30-32). Regarding endometrium, this system has not been studied much in literature. Few studies stated aberrant type expression correlated with type 2 endometrial cancers and in high-grade EEC where it was also associated with poor prognosis (30, 31). Accordingly, the complete absence of staining in our study can be considered an aberrant TP53 expression.

In our study, we found 22% (13/60) of cases expressed CEA as luminal membranous staining and all were morphologically called EEC, grade I on histology. Studies have observed focal luminal membranous CEA staining in mucinous endometrial carcinomas (18, 31, 33). As EEC are usually negative for CEA, combined expression of both EEC and CEA indicates endometrial mucinous carcinoma (22).

CEA is a useful marker when used along with ER, vimentin, and p16 to distinguish between primary endometrial (ER+, CEA+, vimentin+, p16-) and endo cervical adenocarcinomas (CEA+, ER-, vimentin-, p16+) in preoperative biopsy samples (31). The EEC usually does not express or very focally express CEA in the glandular component. 

Napsin A is a well-established marker for clear cell carcinomas in female genital tract cancers of both the ovary and endometrium (10, 28, 34). In the current study, we found a total of 5% of cases (3/60) expressing Napsin A. Of that, 66.6% (2/3) were morphologically low-grade EEC and one was SC with focal clear cell areas. A study by Al Maghrabi* et al.* (35) showed Napsin A expression in 9.4% (5/53) of EEC, 22.2% (2/9) cases of SC and 66.66% of the CCC.

## Conclusion

In the EEC cases, the younger age group will be more likely to express ER. The p53 expression is mostly seen in the elderly age group with larger tumor sizes and high-grade tumors. Complete negative expression of p53 in high-grade tumors needs to be carefully evaluated as it suggests aberrant p53 expression. An inverse association was observed between ER and p53 expression among the cases. The CEA is a valuable factor in identifying EEC with mucinous differentiation. Though Napsin A is traditionally used for clear cell carcinoma, it can be focally positive in EEC. 

Our study underlines the importance of using a panel of markers based on histomorphology to subclassify EC on routine reporting. It also highlights the correlation of these markers with tumor grade, stage, and other clinicopathological prognostic indicators suggesting their role as potential biomarkers. 
